# Acoustofluidic separation of prolate and spherical micro-objects

**DOI:** 10.1038/s41378-023-00636-7

**Published:** 2024-01-11

**Authors:** Muhammad Soban Khan, Mushtaq Ali, Song Ha Lee, Keun Young Jang, Seong Jae Lee, Jinsoo Park

**Affiliations:** 1 Department of Mechanical Engineering, Chonnam National University, 77 Yongbong-ro, Buk-gu, Gwangju, 61186 Republic of Korea; 2 Department of Polymer Engineering, The University of Suwon, 17 Wauan-gil, Bongdam-eup, Hwaseong, Gyeonggi 18323 Republic of Korea

**Keywords:** Engineering, Physics, Chemistry, Materials science

## Abstract

Most microfluidic separation techniques rely largely on object size as a separation marker. The ability to separate micro-objects based on their shape is crucial in various biomedical and chemical assays. Here, we develop an on-demand, label-free acoustofluidic method to separate prolate ellipsoids from spherical microparticles based on traveling surface acoustic wave-induced acoustic radiation force and torque. The freely rotating non-spherical micro-objects were aligned under the progressive acoustic field by the counterrotating radiation torque, and the major axis of the prolate ellipsoids was parallel to the progressive wave propagation. The specific alignment of the ellipsoidal particles resulted in a reduction in the cross-sectional area perpendicular to the wave propagation. As a consequence, the acoustic backscattering decreased, resulting in a decreased magnitude of the radiation force. Through the variation in radiation force, which depended on the micro-object morphology enabled the acoustofluidic shape-based separation. We conducted numerical simulations for the wave scattering of spherical and prolate objects to elucidate the working mechanism underlying the proposed method. A series of experiments with polystyrene microspheres, prolate ellipsoids, and peanut-shaped microparticles were performed for validation. Through quantitative analysis of the separation efficiency, we confirmed the high purity and high recovery rate of the proposed acoustofluidic shape-based separation of micro-objects. As a bioparticle, we utilize *Thalassiosira eccentrica* to perform shape-based separation, as the species has a variety of potential applications in drug delivery, biosensing, nanofabrication, bioencapsulation and immunoisolation.

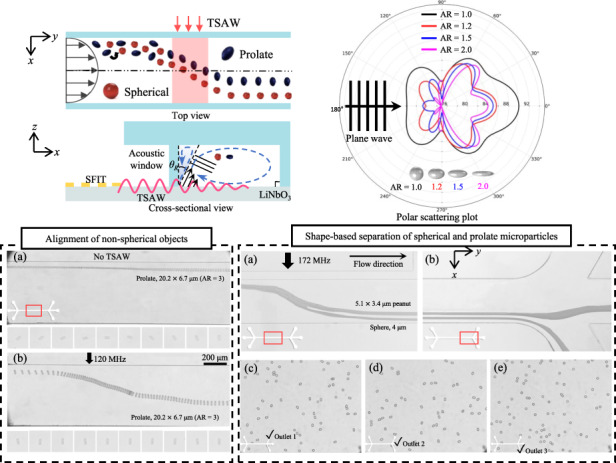

## Introduction

Separation of micro-objects in microfluidics, such as particles and cells, is essential in sample preparation and plays a crucial role in biological, chemical, and biomedical research, as well as in health care diagnostics and environmental applications^1–4^. Microfluidic sample separation can be realized using markers, such as size, label, including magnetic and electric properties, deformability, density, or combinations thereof^5^. Thus far, various active and passive microfluidic techniques have been developed for particle separation^6–9^. Among the passive separation methods, deterministic lateral displacement^1,10,11^ and inertial microfluidic^12–14^ approaches have been widely used to separate micro-objects in a high-throughput manner based on internal hydrodynamic forces. In addition, active separation techniques use external force fields based on dielectrophoresis^15^, magnetophoresis^16,17^, optophoresis^18^, and acoustophoresis^19^. In most passive and active microfluidics sample separation methods, the forces acting on the suspended micro-objects are usually proportional to the size of the micro-objects. Size-based microfluidic sample separation can be achieved since larger micro-objects are subject to forces of larger magnitude. Compared to numerous studies on size-based separation, little effort has been devoted to developing microfluidic shape-based separation techniques.

Shape is an important physical property of micro-objects. Shape-based separation and isolation of particles and cells have attracted interest due to the potential applications of these techniques. For example, cell shape provides useful information for cell synchronization and disease diagnosis, in addition to serving as an indicator of cell growth conditions in many biological samples^20–22^. Li et al. investigated a specific microalga, *Euglena gracilis*, and found that multishaped microalga produced various types of nutrients as a promising alternative to fossil fuels^23^. Barua et al. reported the significance of shape in the specificity of binding and uptake of particulate antibodies and nanoparticles and suggested principles for improved drug delivery^24^. Moreover, *Candida albicans*, which circulates in the bloodstream and is highly pathogenic compared to species of the genus *Candida*, exhibits specific shapes that can be distinguished from those of the genus *Candida*^6^. Similarly, in recent years, yeast has attracted considerable interest as a workhorse in fermentative production^25,26^. In particular, Li et al. reported that a single-celled eukaryotic microbe, *Saccharomyces cerevisiae*, acquires various shapes and is among the most attractive microbial species from the viewpoint of industrial production. Studies of diatoms have a variety of potential applications, including optics, photonics, catalysis, nanofabrication, biosensing, drug delivery, filtration, bioencapsulation and immunoisolation^27^. One specific type of diatom is *Thalassiosira eccentrica*, also known as *T. eccentrica*, a species of diatom that is found in marine environments. This species is known for its unique and distinctive morphology. *T. eccentrica* has a cylindrical or barrel-shaped cell, with one end wider than the other^28^. The word “eccentrica” in its name refers to its eccentric or asymmetrical shape. Despite being the most promising source of biomass, microalgae cultures usually have low concentrations and often contain bacteria, impurities, or other hybrid algal species, which results in a decline in quality^29^ and may cause serious threats to human health in pharmaceutical applications^30^. Therefore, to ensure the safety, efficiency, and high quality of microalgae products, microalgae cultures must be purified and enriched before downstream processing and cultivation^30^.

To date, most of the existing microfluidic sample manipulation methods use size as a separation marker, rather than shape^6,7^. Only a few microfluidic approaches have been proposed to separate micro-object based on their shape. Recent advances in microfluidic shape-based separation include hydrodynamic filtration^12^, deterministic lateral displacement^10,11,13^, inertial microfluidics^31,32^, dielectrophoresis^33^, and magnetophoresis^21^. Passive methods are simple to operate and offer high throughput; however, the methods cannot be operated on demand, and their efficiencies highly depend on fluid properties, flow conditions, and microchannel geometries. On the other hand, the previous active methods require a label as a separation marker, either magnetic or electric, which significantly limits their practical applicability. In this regard, although these aforementioned strategies are promising, there still remains a demand for label-free microfluidic shape-based separation techniques that function in an active manner and can be operated on-demand.

In this study, we propose acoustofluidic shape-based separation of micro-objects based on traveling surface acoustic wave (TSAW)-induced acoustic radiation force (ARF) and torque (ART). We synthesized spherical polystyrene (PS) microspheres and uniaxially stretched them to fabricate prolate microparticles of varying aspect ratios (ARs). We also conducted numerical simulation of acoustic wave scattering to investigate the effects of the micro-object shape on the wave scattering, ARF, and ART. Based on these findings, we performed experiments for acoustofluidic separation of spherical and prolate microparticles of varying aspect ratios and achieved label-free, on-demand, shape-based separation of micro-objects with high purity and recovery rates.

### Methodology

#### Device configuration and working principle

**Fig. 1:**
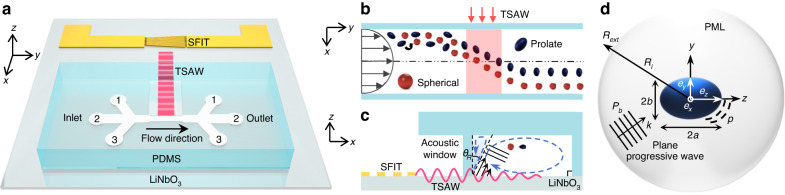
Schematic diagram of an acoustofluidic chip for shape-based separation. **a** A schematic diagram of the proposed acoustofluidic device. **b** Top-view of the midstream microchannel. **c** Cross-sectional view of the midstream microchannel. **d** A rigid ellipsoid modeled system exposed to incident plane progressive waves

The proposed cross-type acoustofluidic device is illustrated in Fig. 1a which consists of a slanted-finger interdigital transducer (SFIT) placed on a piezoelectric lithium niobate (LiNbO_3_) substrate with a polydimethylsiloxane (PDMS) microchannel placed on top of the substrate. The SFIT with linearly varying electrode width and spacing has a resonant frequency bandwidth. When a radio-frequency (RF) alternating current (AC) signal is fed to the SFIT, only the corresponding region, the resonant frequency of which matches the applied frequency, produces TSAWs. By tuning the frequency of the applied AC signal, TSAWs with the corresponding frequency are generated at different locations of the SFIT^34^. Similar to our previous studies, a straight interdigital transducer can be utilized^35–37^. The microchannel has three inlets and three outlets, and the flow direction is perpendicular to the propagation direction of TSAWs produced from the SFIT. The sample fluid flow (polystyrene microparticle solution in DI water) through Inlet 2 was sandwiched by two sheath flows (DI water) through Inlet 1 and Inlet 3. Their flow rates were independently controlled to guide the sample flow near the wall by the SFIT. An acoustic window in the form of an air cavity was introduced in the microchannel to prevent waves from greatly absorbing in the PDMS wall before interactions with fluids occurred inside the microchannel.

Figure 1b shows the top view of the midstream microchannel in which spherical (red) and prolate (blue) PS microparticles suspended in the sample fluid flow were exposed to the plane progressive wave field. To demonstrate the shape-based (not size-based) separation of micro-objects, we fabricated prolate microparticles with varying aspect ratios by uniaxially stretching the spherical particles^38^. The prolate and spherical particles had the same volume but different shapes. Prior to being subjected to the acoustic field, both types of particles rotate while flowing along the microchannel^39^. In the acoustic field, the spherical particles experienced TSAW-induced ARF due to the inhomogeneous wave that scattered off the particles^40,41^. In contrast, interestingly, the microparticles that prolated upon interaction with the TSAWs no longer rotated, and instead, they became aligned and their major axis was parallel to the TSAW propagation. The prolate particle alignment is attributed to TSAW-induced ART^42–44^, in addition to ARF. The ART (${\tau }_{{rad}}$) acting on an ellipsoid can be expressed as ${\tau }_{{rad}}=V{E}_{0}{Q}_{{rad}}\left({e}_{k}\cdot {e}_{z}\right)\left({e}_{k}\times {e}_{z}\right)$, where $V$ is the object volume, ${E}_{0}$ is the characteristic energy density of the incident wave, ${Q}_{{rad}}$ is the dimensionless radiation torque efficiency, ${e}_{k}$ is the unit vector along the wave propagation direction *k*, and ${e}_{z}$ is the unit vector along the orientation *z*^45^. The characteristic energy density of the incident wave is defined as ${E}_{0}={\rho }_{0}{k}^{2}{\varnothing }_{0}^{2}/2$, where ${\rho }_{0}$ is the object density, $k$ is the wavenumber, and ${\varnothing }_{0}$ is the complex amplitude of the velocity potential. The dimensionless radiation torque efficiency ${Q}_{{rad}}$ is defined as ${Q}_{{rad}}=1+3\left({\xi }_{0}+1\right)\left[2+\mathrm{ln}\left(\frac{{\xi }_{0}-1}{2}\right)\right]$, where ${\xi }_{0}$ is the radial distance. The ART experienced by the ellipsoid is determined by the interaction between the incident wave and the ellipsoid orientation, as well as the aspect ratio. Specifically, the ART is primarily influenced by the alignment between the major axis (${e}_{z}$) and the propagation direction of the incident wave (${e}_{k}$). The orientation angle (${\theta }_{k}$) of the wave propagation direction is determined from $\cos {\theta }_{k}={e}_{k}\cdot {e}_{z}$. The ART acting on an ellipsoid exhibits distinct behavior based on the ${\theta }_{k}$ between ${e}_{k}$ and ${e}_{z}$. When the incident wave propagates along the major axis (${\theta }_{k}$ = 0, π), resulting in end-on incidence, and perpendicular to the major axis (${\theta }_{k}$ = π/2), leading to broadside incidence, the ART becomes zero due to the axial symmetry of the ellipsoid and orthogonal alignment with the wave. On the other hand, with the other orientation angles, the ellipsoids in an acoustic field experience ART, resulting in rotation and consequent alignment of the ellipsoids.

Asymmetric wave scattering occurs as prolate particles rotate, unlike spherical particles, and results in counterrotating forces. Clockwise or counterclockwise rotational forces are applied to the prolate particles until symmetric wave scattering occurs, as the major axis of the prolate particles is aligned with the wave propagation direction. The specific orientation of the prolate particles results in a reduced projected surface area for acoustic wave scattering compared to the spherical particles. In particular, a decrease in the backscattering (opposite direction to the incident wave propagation) leads to a decline in ARF, which is the primary driving force for lateral migration of the object in the wave propagation direction. As a result, the spherical and prolate particles can be separated since the prolate microparticles were less deflected than the spherical particles, as depicted from a cross-sectional view in Fig. 1c. As previously reported^37^, a microchannel anechoic corner (MAC) region is formed at the top-left corner of the microchannel due to the refraction of the longitudinal wave at the Rayleigh angle *θ*_*R*_. The sample flow was carefully introduced into the microchannel so that the particles would be located outside the MAC region.

#### Numerical simulation of wave scattering off spherical and prolate particles

Figure 1d depicts a rigid ellipsoid modeled system that was exposed to an incident plane progressive wave, hereinafter called the background pressure field *P*_*b*_, to perform numerical simulation of wave scattering off spherical and prolate particles. In the model, the ellipsoid is located inside a computational domain of radius *R*_*i*_ bounded by the perfectly matched layer (PML)^46^, where *x*, *y*, and *z* are the semiaxes of the ellipsoid, and *p* is the scattered field off the object. The numerical model was constructed and simulated using COMSOL Multiphysics 6.0 to investigate the wave scattering characteristics. The scattered field, through which the incident pressure field can be separated from the dispersed field, is determined at a specific distance of *R*_*ext*_ = 100 μm beyond the computational domain. The total acoustic field *p*_*t*_ is determined as ${p}_{t}={p}_{0}{e}^{-i({\boldsymbol{k}}\cdot x)}+p$ and as the sum of the scattered and background pressure fields, where *p*_*0*_ is the wave amplitude, ***k*** is the wavenumber expressed as ***k*** = 2π*f*_0_/*c*_0_, *f*_*0*_ is the acoustic frequency, and *c*_*0*_ is the speed of sound. For the surrounding fluid, water at 20 °C was considered, as in the experiments presented later.

After the pressure acoustics model was solved, the exterior field calculation feature was used to determine the pressure outside the computational domain. The exterior field calculation feature solved the Helmholtz–Kirchhoff (H-K) integral on the selected boundaries. The selected boundaries needed to form a closed surface around all sources and scatterers. Note that two versions of the H-K integral existed: one that determined only the pressure at the infinity limit and another that solved the entire H-K integral. In this model, we used the full integral and thus could determine the exact exterior field pressure (including phase) at any point and distance outside the computational domain. For plotting purposes, the exterior-field pressure variable *p*_*ext*_ was defined in the model. This variable represented the pressure at any coordinates *x*, *y*, and *z* outside the boundary, on which the exterior-field calculation was defined. The exterior-field pressure and exterior-field sound pressure level were easily plotted and visualized using radiation pattern plot types. To precisely evaluate the exterior-field variable, the H-K integral was evaluated. The normal derivative of the pressure on the exterior-field surface was determined using a single boundary layer mesh handled automatically by the physics-controlled mesh.

#### Fabrication of spherical and prolate microparticles

To validate the proposed acoustofluidic shape-based separation method, we fabricated spherical and prolate PS microparticles. First, monodispersed spherical PS microparticles were synthesized for the seed microspheres by the emulsifier-free emulsion polymerization method (details can be found in our previous study)^38^. The resulting seed PS microspheres were used to fabricate prolate particles by uniaxial stretching, which is among the most suitable techniques for producing homogeneous prolate particles. Briefly, 15 g of poly(vinyl alcohol) (PVA) was dissolved in 150 mL of distilled water at 80 °C for 4–5 h with a magnetic stirrer. The aqueous PVA solution was cooled to room temperature, and 0.1 wt% PS seed microspheres were added to the solution. The homogeneously dispersed mixture was then poured into an aluminum tray, in which water evaporated slowly at room temperature. The flexible PVA film containing PS microspheres was formed after drying for 36–48 h. The film was cut into 2 $\times$ 8 cm^2^ rectangular patches for the uniaxial stretching process to prepare prolate spheroids. The chamber temperature was set to 135 °C and film stretching was performed by securely fastening these PVA films to the tensile grips of a universal testing machine (UTM) to prepare prolate spheroids at a rate of 0.5 mm/s with a 50–300% elongation range, depending on the desired aspect ratio of the prolate microparticles. The film was stretched, cooled down and then washed to collect the fabricated prolate particles. In this collection process, washing and vortex mixing were repeated at 80 °C to efficiently remove the PVA matrix, followed by centrifugation. The detailed procedures used to fabricate the spherical and prolate particles can be found in our previous study^38^.

**Fig. 2:**
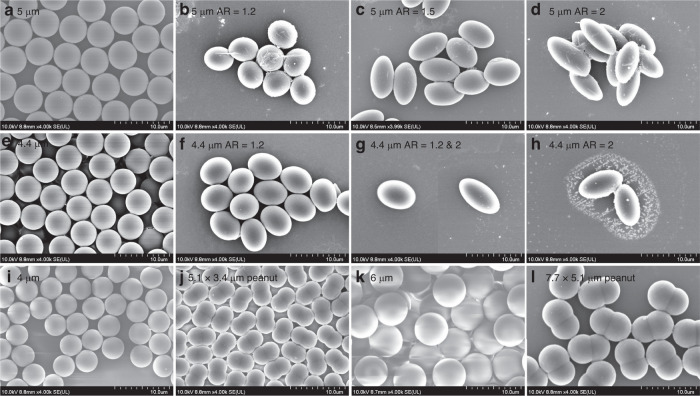
SEM images of all the particles used in this study. **a** 5 μm PS microspheres with volume (V) = 65.45 μm^3^. Prolate ellipsoidal PS microparticles fabricated from the 5 μm microspheres with aspect ratio of **b** 1.2 with V = 64.332 μm^3^, **c** 1.5 with V = 64.74 μm^3^, and **d** 2 with V = 65.21 μm^3^. **e** 4.4 μm PS microspheres with V = 44.602 μm^3^. Prolate ellipsoidal PS microparticles fabricated from the 4.4 μm microspheres with aspect ratio of **f** 1.2 with V = 43.092 μm^3^, **g** 1.2 and 2, and **h** 2 with V = 43.59 μm^3^. **i** 4 μm PS microspheres with V = 33.51 μm^3^. **j** 5.1 $\times$ 3.4 μm peanut-shaped PS particles with V = 34 μm^3^. **k** 6 μm PS microspheres with V = 113 μm^3^. **l** 7.7 $\times$ 5.1 μm peanut-shaped PS particles with V = 117 μm^3^

Figure 2 shows the SEM images of all PS microparticles, including spherical, prolate and peanut-shaped PS microparticles, used in the validation experiments. Figure 2a–d shows spherical PS particles 5 µm in diameter and their derivative prolate particles with aspect ratios of 1.2, 1.5, and 2, respectively. Figure 2e–h shows spherical PS particles 4.4 µm in diameter and their derivative prolate particles with ARs of 1.2, and 2, respectively. Figure 2g represents a side-by-side comparison of the two prolate PS microparticles with different AR values of 1.2 and 2. Note that the volume (*V*) of the microparticles in Fig. 2a–d and Fig. 2e–h remained the same while their shape was modified. Figure 2i, j shows the 4 µm spherical PS microparticles and 5.1 $\times$ 3.4 µm peanut-shaped PS particles, respectively. Figure 2k, l shows the 6 µm spherical PS microparticles and 7.7 $\times$ 5.1 µm peanut-shaped PS particles, respectively. Note that the PS microparticles in Fig. 2i–l were commercial products, unlike the fabricated microparticles in Fig. 2a–h. The aspect ratios of the PS microparticles were measured by processing the SEM images using ImageJ. The statistics of the particle dimensions for each particle type are included in Supplementary Fig. S1a in the Supplementary Material. Moreover, for clarification, we summarized the volume of all particles used in Supplementary Table S1 in the Supplementary Materials.

#### Cell culture

The *T. eccentrica* (LIMS-PS-1759) sample was obtained from the Korea Institute of Ocean Science & Technology (KIOST). Before experiments were performed, microalgae were cultured in 50 mL conical flasks at room temperature using saltwater culture media (f/2) under continuous illumination of a 3000-lux intensity lamp and shaken at 150 rpm. A cultured algae sample of 1 mL was taken in a microcentrifuge tube (Hyundai Micro) and subjected to centrifugation at 1500 rpm for 5 min using a mini centrifuge (Daihan Scientific). After centrifugation, the microalgae were resuspended in fresh growth media and diluted to the desired cell concentration for shape-based separation.

### Experimental

A pair of slanted-finger electrodes composed of a bimetallic layer of Cr and Au (thickness of 20 and 100 nm, respectively) were deposited to form an SFIT on a 500-μm-thick 128°-rotated, Y-cut, X-propagating LiNbO_3_ substrate (MTI Korea) through photolithography, E-beam evaporation, and lift-off procedures^47^. The two types of SFITs utilized in the experiments had varying electrode widths and spacings (*λ*/4) of 6.5–8.5 μm and 5–7 μm (metal ratio of 0.5), respectively. Both SFITs contained 40 finger pairs and had a total aperture of 1 mm. A vector network analyzer (E5071B, Agilent Technologies) was used to determine the resonant frequency bandwidths of the SFITs as 116–152 MHz and 141–198 MHz. An RF signal generator (BSG F10, Belektronig GmbH) was used to apply RF AC signals to the SFIT. For rectangular PDMS microchannel fabrication, the PR mold was treated with 1H, 1H, 2H, and 2H-perfluorooctyltriethoxysilane (Sigma‒Aldrich) before the PDMS mixture (Sylgard 184 A and 184B, Dow Corning) was poured on it. Thereafter, oxygen plasma (Covance, Femto Science) was applied to permanently attach the fabricated microchannel to the LiNbO_3_ substrate. The height (*h*) and width (*w*) of the rectangular microchannel were 67 µm and 500 µm, respectively. Fluorescent PS microspheres (Thermo Scientific, Inc.) with diameters of 4 and 6 μm were utilized, the *V* of which was almost identical to that of their corresponding peanut-shaped particles (Magsphere, Inc.). A syringe pump (neMESYS Cetoni GmbH) was used to inject the sample and sheath fluid flows into the microchannels. The microparticles were suspended in distilled water (Dyne Bio Inc.) and 5 wt% TWEEN® 20 (Sigma‒Aldrich). A high-speed complementary metal-oxide semiconductor (CMOS) camera (VEO 710 L, Phantom) and an inverted microscope (IX73, Olympus) were used to investigate the microparticles’ behaviors.

## Results and discussion

### Acoustic wave scattering depending on shape and its effects on ARF and ART

The TSAW-induced ARF and ART play a significant role in the proposed acoustofluidic shape-based separation of micro-objects. The Rayleigh-type surface waves propagate only along the piezoelectric substrate and refract into the microchannel in the form of longitudinal waves in the fluid, as shown in Fig. 1c. As the refracted waves interact with the spherical and prolate microparticles in the fluid, inhomogeneous acoustic wave scattering off both types of microparticles occurs; as a result, TSAW-induced ARF and ART acts on the suspended objects^45,48^. To elucidate the underlying physics behind the difference in the lateral migration of the spherical and prolate micro-objects in Fig. 1b, we conducted numerical simulation of the acoustic wave scattering using the numerical scheme depicted in Fig. 1d.

**Fig. 3:**
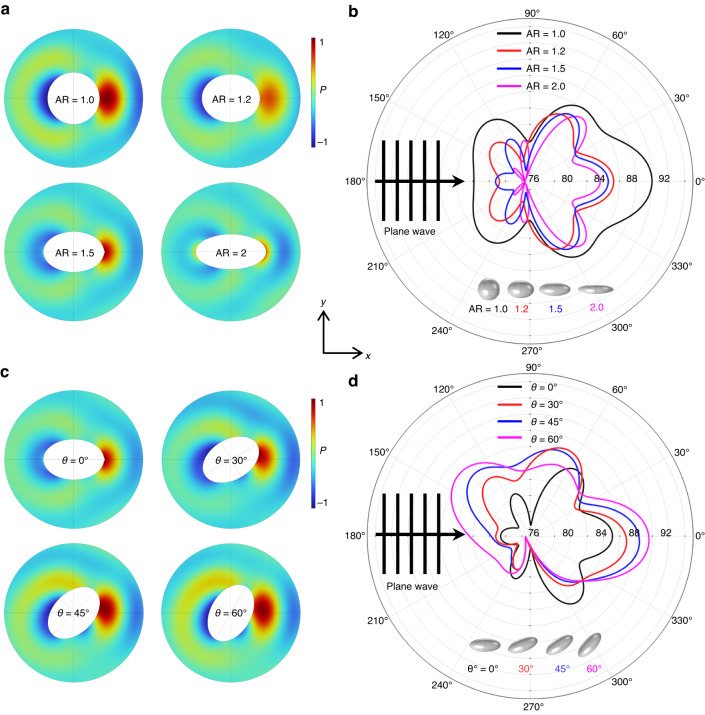
Polar scattering plots from numerical simulation of wave scattering off spherical and prolate ellipsoids. Wave scattering off spherical (black) and prolate objects with aspect ratio of 1.2 (red), 1.5 (blue), and 2.0 (magenta) in the **a** scattering field and **b** external field. Wave scattering off prolate ellipsoids with aspect ratio of 1.5 at varying orientation angle θ = 0° (black), 30° (red), 45° (blue), and 60° (magenta) in the **c** scattering field and **d** external field

Figure 3a describes the scattering field off the object, and the incident waves were assumed to be plane progressive waves that propagate from left (180°) to right (0°) in the *x*-direction with a frequency of 141 MHz. The Helmholtz number was approximately 1.5^49^, indicating Mie scattering^50–52^. This alignment was attributed to the TSAW-induced counterrotating torque exerted on the rotating nonspherical particles^42^. Among the several particle aspect ratios evaluated, particles with AR = 1 exhibited the largest forward and backward scattering magnitudes at θ = 0° and 180°, respectively. As we increased the aspect ratio of the particles, the forward and backward scattering magnitude decreased. Figure 3b shows the acoustic pressure level in the external field at a distance of *R*_*ext*_ = 100 μm for the 5 μm microspheres (black) and their derivative prolate microparticles with aspect ratios of 1.2 (red), 1.5 (blue) and 2.0 (magenta) of the same *V* as the microspheres. In the polar scattering plot, the scattering can be categorized into forward-, backward-, and side-scattering in the directions of 0°, 180°, and 90 and 270°, respectively^53^. The lateral migration of the micro-objects occurs in the direction of the TSAW propagation due to the ARF, the magnitude of which depends primarily on the backscattering. Mitri proposed a theoretical model for the ARF acting on a two-dimensional infinitely long elliptical cylinder exposed to plane progressive waves^53^. We assumed that the proposed acoustofluidic phenomena of three-dimensional prolate elliptical micro-objects can be interpreted since the prolate micro-objects primarily rotated in the *xy*-plane in the so-called tumbling mode of rotation^39^, as will be presented in the subsequent section. The radiation force function ($${Y}_{p})$$ of an object within an acoustic field can be expressed by $${Y}_{p}=\left(a/{a}_{{eff}}\right)\frac{1}{4}{\int }_{0}^{2\pi }{\left|{f}_{\infty }\left(k,\theta \right)\right|}^{2}(1-\cos \theta )d\theta$$ where $$a$$ is the object radius in the major axis, $${a}_{{eff}}=\sqrt{({a}^{2}+{b}^{2})/2}$$ is the effective radius, $$b$$ is the object radius in the minor axis, and $$\left|{f}_{\infty }=\left(k,\theta \right)\right|$$ is the magnitude of the backscattering. The ARF function characterizes the ARF exerted on the object by the incident acoustic wave. The ARF arises due to the momentum transfer from the incident wave to the object, which results from scattering and interference effects. The backscattering term $$\left|{f}_{\infty }=\left(k,\theta \right)\right|$$ is directly proportional to the ARF because a higher magnitude of backscattering implies that the momentum transfer from the incident wave to the object is more significant, resulting in a greater magnitude of the ARF^43,53^.

The backscattering is proportional to the cross-sectional area (perpendicular to the wave propagation) of the scatterer in the acoustic field^53^. An increase in backscattering results in increased momentum transfer from the incident waves to the scatterer, leading to an increase in lateral migration in the wave propagation direction. The prolate particles of the same *V* as the spherical particles have a reduced cross-sectional area when their major axis is aligned in the wave propagation direction.

The acoustic backscattering level can be regarded as the scattering projected in the backward direction (180°) from 90° to 270°^54^. As determined by our calculation, the backscattering off the microspheres (AR = 1) was greater than that of the prolate ellipsoids. Additionally, the backscattering of the ellipsoids decreased with increasing aspect ratio. Sidelobes were observed in the polar plots, the effects of which were presumably negligible on the ARF^55–57^. This difference in the acoustic backscattering suggests that the magnitude of the TSAW-induced ARF could vary depending on the shape of the micro-objects, which will be experimentally validated later.

We performed numerical simulations of acoustic wave scattering off the prolate ellipsoid with the aspect ratio of 1.5, as shown in Fig. 3c, d. The scattering field was plotted for the prolate objects with varying orientations with respect to wave propagations of *θ* = 0° (black), 30° (red), 45° (blue), and 60° (magenta) (Fig. 3c). All the parameters were set identically to those in Fig. 3a unless otherwise specified. The side scattering off the ellipsoid of θ = 0°^58^, aligned parallel to the wave propagation, was symmetric in the directions of 90° and 270°. The symmetric side scattering suggests that TSAW-induced ART is not generated in the aligned prolate ellipsoids. On the other hand, as the ellipsoids were misaligned with the wave propagation, the two components of the side scattering were imbalanced due to the shape asymmetry^59^. Due to the counterclockwise orientation of the ellipsoids, the side scattering toward 90° was greater than that toward 270° in all cases of *θ* = 0°, 30°, 45°, and 60°. The side scattering toward 270° with greater magnitudes indicates the clockwise torque acting on the counterclockwise-rotated ellipsoids with respect to the wave propagation. The acoustic pressure level in the external field at a distance of *R*_*ext*_ = 100 μm was also plotted (Fig. 3d). From the simulation results, we found that the imbalance in the side scattering gradually increased with increasing orientation angle of *θ* = 30–60°, leading to an increase in the magnitude of the counterrotating torque exerted on the ellipsoids.

### Acoustofluidic shape-based separation of spherical and prolate microparticles

**Fig. 4:**
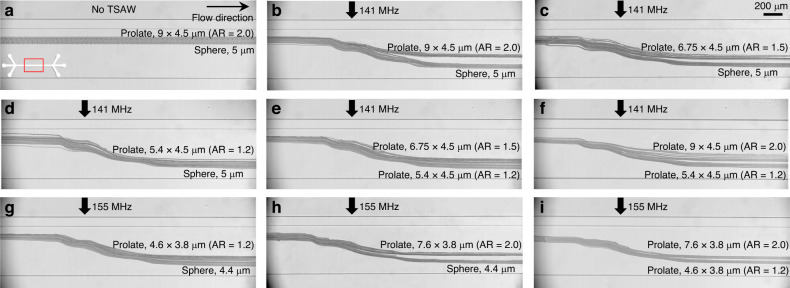
Microscopy images of acoustofluidic shape-based separation of spherical and prolate PS microparticles. **a** 5 μm spheres with volume (V) of 65.45 μm^3^ and 9 $\times$ 4.5 μm (AR = 2.0) prolate particles with V = 65.21 μm^3^ without acoustic field. **b** 5 μm spheres and 9 $\times$ 4.5 μm (AR = 2.0) prolate particles with the 141 MHz acoustic field. **c** 5 μm spheres and 6.75 $\times$ 4.5 μm (AR = 1.5) prolate particles with V = 64.74 μm^3^ at a 141 MHz acoustic field. **d** 5 μm spheres and 5.4 $\times$ 4.5 μm (AR = 1.2) prolate particles with V = 64.332 μm^3^ at a 141 MHz acoustic field. **e** 6.75 $\times$ 4.5 μm (AR = 1.5) and 5.4 $\times$ 4.5 μm (AR = 1.2) prolate particles with the 141 MHz acoustic field. **f** 9 $\times$ 4.5 μm (AR = 2.0) and 5.4 $\times$ 4.5 μm (AR = 1.2) prolate particles with the 141 MHz acoustic field. **g** 4.4 μm spheres with V = 44.602 μm^3^ and 4.6 $\times$ 3.8 μm (AR = 1.2) prolate particles with V = 43.092 μm^3^ at a 155 MHz acoustic field. **h** 4.4 μm spheres and 7.6 $\times$ 3.8 μm (AR = 2.0) prolate particles with V = 43.59 μm^3^ at a 155 MHz acoustic field. **i** 7.6 $\times$ 3.8 μm (AR = 2.0) and 4.6 $\times$ 3.8 μm (AR = 1.2) prolate particles with the 155 MHz acoustic field. Scale bar: 200 μm

The ARF is a nonlinear function of size in the Mie scattering regime in which the object size is comparable to the acoustic wavelength or larger^51^. The elastic sphere theory has been widely used to estimate the ARF acting on a spheroid and to determine the optimal frequency for acoustofluidic manipulation^41^. However, the elastic sphere theory cannot be used on nonspherical objects, such as the prolate ellipsoids used in our study. The dimensionless ARF function (${Y}_{p}$) that acts on nonspherical objects can be expressed as ${Y}_{p}=\frac{\left\langle F\right\rangle }{\pi {({a}^{2}/{a}_{{eff}})}^{2}{{\rm{E}}}_{0}\,}$, where $\left\langle F\right\rangle$ is the time-averaged ARF, *a* is the object radius in the major axis, ${a}_{{eff}}=\sqrt{({a}^{2}+{b}^{2})/2}$ is the effective radius, $b$ is the object radius in the minor axis, and ${E}_{0}$ is the characteristic energy density of the incident wave, which is defined as ${{\rm{E}}}_{0}={\rho }_{0}{k}^{2}{\varnothing }_{0}^{2}/2$, where ${\rho }_{0}$ is the object density, $k$ is the wavenumber, and ${\varnothing }_{0}$ is the complex amplitude of the velocity potential. As in the equation above and demonstrated in our experiments in Fig. 4, the prolate ellipsoidal microparticles of varying aspect ratio (*a/b*), thus varying effective radius, experienced different magnitudes of the ARF, especially when they were aligned along the wave propagation by the acoustic radiation torque.

For experimental validation of the findings obtained from the numerical simulation in Fig. 3, we conducted a series of particle separation experiments using the PS microspheres and prolate ellipsoids of varying aspect ratios that we fabricated (Fig. 2). Figure 4 shows stacked microscopy images in the midstream microchannel (67 µm in height and 500 µm in width), marked with a red box in Fig. 4a, in which the suspended microparticles were exposed to the acoustic waves propagating perpendicular to the flow direction. The frequency of the TSAWs was carefully chosen for the most energy-efficient PS particle manipulation with a Helmholtz number of 1.5 for 5 μm at 141 MHz and 1.44 for 4.4 μm at 155 MHz^37^. The two sheath fluid flows were introduced at volumetric flow rates of 30 and 200 µL/h through Inlets 1 and 3, respectively, to narrowly focus the sample flow at 20 µL/h through Inlet 2 in the vicinity of the sidewall close to the transducer. A 5 wt% surfactant TWEEN® 20 was added to the sample fluid to ensure a stable suspension and reduce the aggregation in the particle solution.

Figure 4a shows the trajectories of the 5 μm PS spheres with *V* = 65.45 μm^3^ without an acoustic field compared to 9 $\times$ 4.5 μm (AR = 2.0) PS prolate ellipsoids with *V* = 65.21 μm^3^ fabricated from the seed microspheres. Due to the absence of TSAW-induced ARF and ART, the two types of micro-objects had almost the same trajectories without any lateral migration. Figure 4b–f demonstrate the trajectories of the 5 μm spheres and 9 $\times$ 4.5 μm (AR = 2.0) prolate ellipsoids, 5 μm spheres and 6.75 $\times$ 4.5 μm (AR = 1.5) prolate ellipsoids with *V* = 64.74 μm^3^, 5 μm spheres and 5.4 $\times$ 4.5 μm (AR = 1.2) prolate ellipsoids with *V* = 64.332 μm^3^, 6.75 $\times$ 4.5 μm (AR = 1.5) and 5.4 $\times$ 4.5 μm (AR = 1.2) prolate ellipsoids, 9.45 $\times$ 4.5 μm (AR = 2.0) and 5.4 $\times$ 4.5 μm (AR = 1.2) prolate ellipsoids with the 141 MHz TSAWs at 6.58 mW, respectively. Relative to the lateral migration of the 5 μm microspheres, the ellipsoidal microparticles showed less displacement. With increasing ARs of the ellipsoidal particles, their lateral migrations decreased, which corresponded with the simulation results in Fig. 3a. The reduced cross-sectional area that was perpendicular to the wave propagation with an increase in aspect ratio caused the decreased TSAW-induced ARF to act on the prolate ellipsoids. We also found that prolate PS microparticles of AR = 1.2 had similar trajectories to the microspheres due to a small difference in the backscattering and were difficult to separate from the microspheres (Fig. 4d), compared to the ellipsoids of AR = 2.0 (Fig. 4b) and AR = 1.5 (Fig. 4c). Figure 4e, f shows the trajectories under the 141 MHz TSAWs at 5.28 mW of the ellipsoidal PS particles of AR = 1.2 and 1.5 and 1.2 and 2.0, respectively. The difference in AR (0.3) in Fig. 4e resulted in almost similar trajectories to the prolate particles, as shown in Fig. 4d, with an AR difference of 0.2. In contrast, Fig. 4f demonstrates that the two types of prolate ellipsoids with an AR difference of 0.8 showed distinct trajectories. The trajectories under the 155 MHz TSAWs (Helmholtz number of 1.5) of the 4.4 μm PS microspheres with *V* = 44.602 μm^3^ and their derivative prolate particles with aspect ratios of 1.2 and 2.0 and *V* values of 43.092 and 43.332 μm^3^, respectively, are presented in Fig. 4g–i. Similar to previous results, the lateral migration difference was observed to increase with increasing aspect ratio difference.

**Fig. 5:**
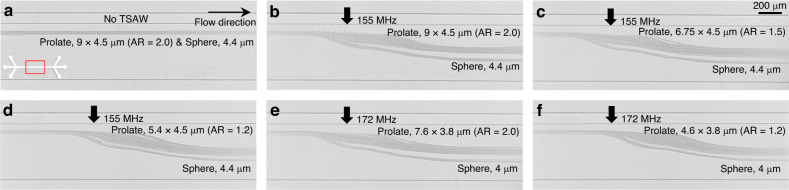
Microscopy images of acoustofluidic shape-based separation of spherical and prolate PS microparticles. **a** 4.4 μm spheres with V = 44.6 μm^3^ and 9 $\times$ 4.5 μm (AR = 2.0) prolate particles with V = 65.21 μm^3^ without acoustic field. Under the effects of TSAW at 155 MHz, **b** 4.4 μm spheres and 9 $\times$ 4.5 μm (AR = 2.0) prolate particles, **c** 4.4 μm spheres and 6.75 $\times$ 4.5 μm (AR = 1.5) prolate particles with V = 64.74 μm^3^, **d** 4.4 μm spheres and 5.4 $\times$ 4.5 μm (AR = 1.2) prolate particles with V = 64.33 μm^3^. Under the effects of TSAW at 172 MHz, **e** 4 μm spheres with V = 33.51 μm^3^ and 7.6 $\times$ 3.8 μm (AR = 2.0) prolate particles with V = 43.59 μm^3^, **f** 4 μm spheres and 4.6 $\times$ 3.8 μm (AR = 1.2) prolate particles with V = 43.092 μm^3^

Figure 4 shows that the spherical and prolate microparticles can be separated in the progressive acoustic field since they are subjected to ARF with different magnitudes depending on the AR. In these experiments, we intentionally used various-shaped particles of the same volume to disregard the size effect on the ARF. In addition, we conducted experiments with microparticles of different sizes and ARs, as shown in Fig. 5. In Fig. 5a–d, we used 9 $\times$ 4.5 μm prolate ellipsoids with ARs of 2.0 (Fig. 5b), 1.5 (Fig. 5c), and 1.2 (Fig. 5d), the volume of which was approximately 65.21 μm^3^, corresponding to an effective diameter of 5 μm, and 4.4 μm spheroids, the volume of which was approximately 44.6 μm^3^. As seen in the figures, even though the elliptical micro-objects were larger in size, the lateral migration of the prolate microparticles was smaller than that of the spherical particles at *f* = 155 MHz. Similar results were also found in Fig. 5e, f, in which we utilized 7.6 $\times$ 3.8 μm prolate ellipsoids with ARs of 2.0 (Fig. 5e) and 2.0 (Fig. 5f), the volume of which was approximately 43 μm^3^, corresponding to an effective diameter of 4.4 μm, and 4 μm spheroids, the volume of which was approximately 33.51 μm^3^. Despite the larger volume, the ARF-induced lateral migration of the prolate ellipsoids was smaller because the magnitude of the ARF was smaller than that of the smaller spheroid particles. These results suggest that the shape effect can be more significant than the size effect in acoustofluidic separation of samples of similar volume.

### Acoustofluidic shape-based separation of spherical and peanut-shaped microparticles

**Fig. 6:**
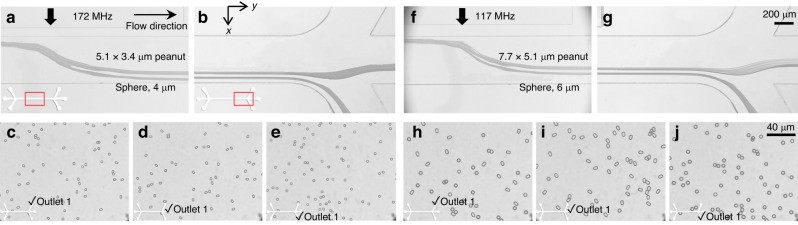
Microscopy images showing the acoustofluidic shape-based separation of spherical and peanut-shaped PS microparticles. 4 μm spheres with volume (V) of 33.51 μm^3^ and 5.1 $\times$ 3.4 μm peanut-shaped microparticles with V of 34 μm^3^ at a 172 MHz acoustic field **a** in the midstream and **b** at the downstream trifurcation. **c** Collected particles from Outlet 1. Collected particles from **d** Outlet 2 and **e** Outlet 3. 6 μm spheres with V of 113 μm^3^ and 7.7 $\times$ 5.1 μm peanut-shaped microparticles with V of 117 μm^3^ at a 117 MHz acoustic field **f** in the midstream and **g** at the downstream trifurcation. **h** Collected particles from Outlet 1. Collected particles from **i** Outlet 2 and **j** Outlet 3. Scale bar: 200 μm

In addition to the prolate ellipsoids, we further performed experiments for acoustofluidic separation of peanut-shaped PS microparticles from microspheres. Yeast cells have attracted significant attention because they can be utilized for biofuel production and pharmaceuticals^24^. Depending on morphology, yeast cells can be largely categorized into singlets, doublets, and clusters, which exhibit different cell characteristics and behaviors^26^. In particular, the doublet cells can be modeled as peanut-shaped objects, which look like two microspheres that partially overlap. In our experiments, we used two types of peanut-shaped PS microparticles with dimensions of 5.1 $\times$ 3.4 μm and 7.7 $\times$ 5.1 μm with *V* values of 34 and 117 μm^3^, respectively. For comparison, 4 and 6 μm fluorescent PS microspheres were used since their *V* was almost identical to their corresponding peanut-shaped particles, 33.51 and 113 μm^3^, respectively. Unlike the monochromic microspheres and ellipsoidal particles used in Fig. 4, we could also perform a quantitative evaluation on the separation efficiency for the fluorescent microspheres and the monochromic peanut-shaped particles. All the experimental conditions remained the same as those in Fig. 6 unless otherwise specified.

Figure 6a–e shows the results of acoustofluidic separation of the 5.1 $\times$ 3.4 μm peanut-shaped particles from 4 μm spherical particles using the 172 MHz TSAWs (Helmholtz number of 1.46) at 6 mW. Figure 6a and b shows the stacked microscopy images in the midstream microchannel and downstream trifurcation connected to three different outlets, respectively. The trajectories of the 5.1 $\times$ 3.4 μm peanut-shaped particles were distinct from those of the 4 μm spherical particles. Despite the morphological differences, the peanut-shaped particles could be approximated as prolate ellipsoids of AR = 1.5. As discussed earlier, the two types of micro-objects could exhibit different particle trajectories when subject to the TSAW field due to the aspect ratio difference of 0.5. As the rotating peanut-shaped microparticles were aligned in the TSAW field due to the counterrotating torque, the major axis of the peanut-shaped particles was tilted parallel to the wave propagation. This specific orientation of the nonspherical objects led to the reduced projected area for the acoustic backscattering, resulting in a consequent decrease in the TSAW-induced ARF, as depicted in Fig. 3. As shown in Fig. 6b, two particle solution streams can be collected at separate outlets. Figure 6c shows the mixture of the 5.1 $\times$ 3.4 μm peanut-shaped and 4 μm spherical particles collected from Outlet 1 without the acoustic field. Figure 6d, e shows the collected particles after acoustofluidic shape-based separation from Outlet 2 and Outlet 3, respectively. In Fig. 6d, most microparticles were found to be peanut-shaped particles due to their smaller lateral migration. In contrast, in Fig. 6e, most of the collected particles were verified as microspheres since they experienced the TSAW-induced ARF with greater magnitude compared to the nonspherical particles. Similar results were obtained from the experiments with the 7.7 $\times$ 5.1 μm peanut-shaped particles from the 6 μm microspheres under the 117 MHz TSAWs (Helmholtz number of 1.49) at 6.23 mW, as in Fig. 6f–j.

**Fig. 7:**
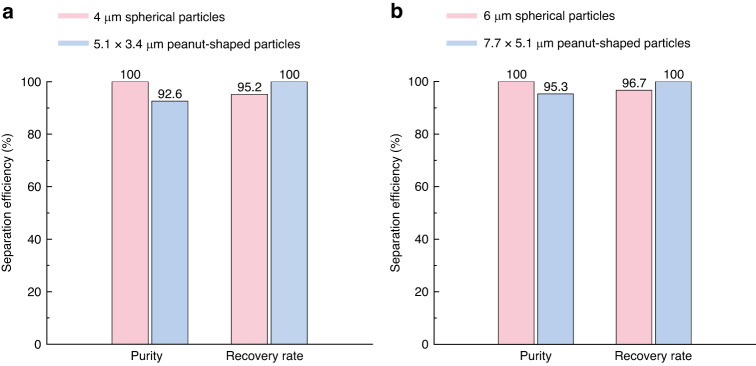
Quantitative evaluation of separation efficiency of the proposed acoustofluidic shape-based separation. **a** Purity and recovery rate of 4 μm spherical (red) and 5.1 $\times$ 3.4 μm peanut-shaped (blue) PS particles. **b** Purity and recovery rate of 6 μm spherical (red) and 7.7 $\times$ 5.1 μm peanut-shaped (blue) PS particles

To quantitatively investigate the proposed acoustofluidic shape-based separation, we processed images of the collected fluorescent spherical and monochromic peanut-shaped particles collected from different outlets in the experiments in Fig. 6. The identified particles with circularity above 0.9 were counted as microspheres, whereas the other particles with circularity below the threshold were regarded as peanut-shaped particles using ImageJ. Figure 7a, b shows the purity and recovery rate for 4 μm spherical (red) and 5.1 $\times$ 3.4 μm peanut-shaped (blue) particles and for 6 μm spherical (red) and 7.7 $\times$ 5.1 μm peanut-shaped (blue) particles, respectively. The purity was calculated as a ratio (the number of target particles at the target outlet to the total number of particles found at the target outlet), and the recovery rate was calculated with a different ratio (the target particles at the target outlet to the total number of particles collected at all outlets combined). The purity and recovery rate of the 4 μm spherical particles were 100% and 95.2%, respectively, while the purity and recovery rate of the 5.1 × 3.4 μm peanut-shaped particles were 92.6% and 100%, respectively. Similarly, 6 μm spherical particles were found to have 100% purity and a 96.7% recovery rate, while the 7.7 × 5.1 μm peanut-shaped particles exhibited a purity of 95.3% and a recovery rate of 100%. Overall, the proposed acoustofluidic shape-based separation was highly efficient regardless of the target micro-object size, provided that the acoustic field with an appropriate frequency was applied, as discussed in our previous studies^37,49,60^.

### Acoustofluidic alignment of non-spherical micro-objects

**Fig. 8:**
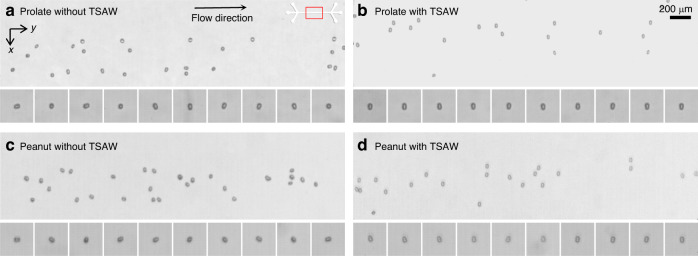
Microscopic snapshot images of acoustofluidic alignment of nonspherical microparticles. 6.75 $\times$ 4.5 μm prolate PS particles **a** without and **b** with acoustic field at 141 MHz. 7.7 $\times$ 5.1 μm peanut-shaped PS particles **c** without and **d** with acoustic field at 117 MHz

The proposed acoustofluidic shape-based separation based on the TSAW-induced ARF and ART originates from the alignment of the non-spherical micro-objects with the progressive wave propagation in the acoustic field. In this regard, we further analyzed the nonspherical particle behaviors under the TSAW field in Fig. 8 (see also Supplementary Movie S1 in the Supporting Information). Figure 8a, b shows microscopic snapshots of the 6.75 $\times$ 4.5 μm (AR = 1.5) prolate ellipsoids without and with the TSAW field at 141 MHz, respectively. In the absence of the acoustic field, the rod-like prolate PS microparticles were either tumbling, rolling, or moving with kayaking patterns while migrating along with the flow^39^. The magnified views of the particles show the rotating ellipsoidal microparticles at varying orientations. On the other hand, under the TSAW field, the major axis of the prolate particles was clearly confirmed to be aligned in the *x*-propagating progressive acoustic waves in the midstream^49^ microchannel. Similarly, Fig. 8c, d shows microscopic snapshots of the 7.7 $\times$ 5.1 μm peanut-shaped PS particles without and with the TSAW field at 117 MHz, respectively. As observed for the prolate ellipsoids, the peanut-shaped microparticles freely rotated along with the flow. In contrast, under exposure to the TSAW field, all peanut-shaped particles were aligned by TSAW-induced ART such that their major axis matched the direction of progressive wave propagation. The specific alignment of the non-spherical micro-objects caused the particles to exhibit a reduced cross-sectional area perpendicular to the TSAWs, leading to the reduced magnitude of the TSAW-induced ARF. Through the variation in the ARF magnitude, which depended on the micro-object shape, the proposed acoustofluidic shape-based separation method could be experimentally validated, as shown in Figs. 4 and 5.

### Acoustofluidic shape-based separation of microalgae

**Fig. 9:**
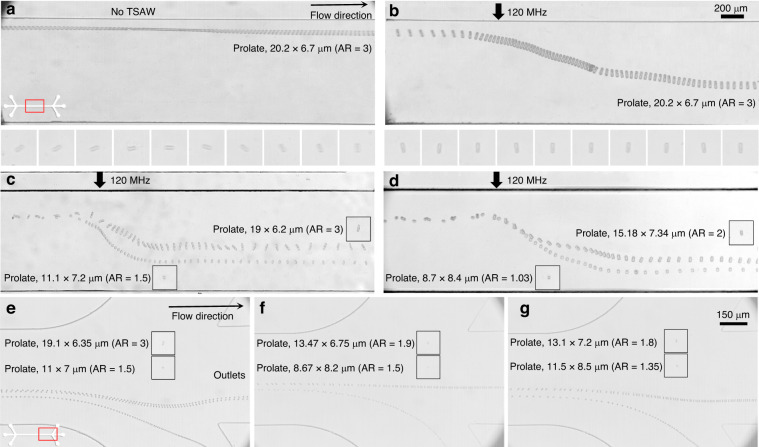
Microscopic snapshot images of acoustofluidic alignment of T. eccentrica throughout the microchannel. **a** with-out acoustic field and **b** with acoustic field. **c**–**g** shows the shape-based separation with T. eccentrica of different aspect ratios. Shape-based separation of T. eccentrica highlighting the outlets of the microchannel (**e**) cells with AR = 3 and 1.5, (**f**) cells with AR = 1.9 and 1.5, and (**g**) cells with AR = 2 and 1.35

*T. eccentrica* is a cylindrical or barrel-shaped cell, with one end wider than the other. The ability to separate the particles will have a variety of potential applications, including applications in optics, photonics, catalysis, nanofabrication, biosensing, drug delivery, filtration, bioencapsulations and immunoisolations^27^. The size distribution of cultured microalgae was measured, as shown in Supplementary Fig. S1b in the Supplementary Material. The results indicated that *T. eccentrica* exhibited distinct shapes that are not randomly scattered; instead, the shapes were associated with particular types of ARs that can be classified (AR $\approx$ 1, 1.1, 1.5, 1.9, 2.0, and 3.0). We investigated the acoustofluidic phenomena related to ART and ARF using prolate bioparticles and applied the proposed method for the shape-based separation of *T. eccentrica*. The experimental conditions for the microalgae separation remained the same as those in the spherical and prolate particle separation, unless specified otherwise. *T. eccentrica* were injected through Inlet 2, while the sheath fluids were introduced through Inlets 1 and 3. *T. eccentrica* were focused near the sidewall to examine the influence of TSAW. Figure 9a, b shows the alignment of *T. eccentrica* with TSAW off and on, respectively, in the 120 MHz acoustic field. The prolate microalgae were observed to be freely tumbling while flowing through the microchannel without any acoustic field (Fig. 9a). In contrast, once the plane progressive acoustic field was applied (Fig. 9b), the prolate microalgae rotated due to the ART, and their major axis was aligned with the wave propagation direction while being deflected by the ARF. These results were consistent with the prolate and peanut-shaped microparticle alignment shown in Fig. 8. As demonstrated in the numerical calculation for wave scattering at varying orientations in Fig. 3d, asymmetric side scattering caused the prolate microalgae to rotate by ART.

Figure 9c demonstrates the trajectories of the 19 $\times$ 6.2 μm (AR = 3.0) and 11.1 $\times$ 7.2 μm (AR = 1.5) *T. eccentrica*, while Fig. 9d represents the trajectories of the 15.18 $\times$ 7.34 μm (AR = 2.0) and 8.7 $\times$ 8.4 μm (AR = 1.03) *T. eccentrica* with the 120 MHz TSAWs at 10.04 mW. In these results, the larger-sized *T. eccentrica* with higher ARs were deflected less in comparison to the smaller-sized *T. eccentrica* with lower ARs. This observation aligns with the findings obtained for the polymer particles of different sizes and ARs, as presented in Fig. 5. These results suggest that the reduction in the cross-sectional area perpendicular to the wave propagation, stemming from increased AR, led to a decrease in the ARF induced by TSAWs on *T. eccentrica* with higher AR. Figure 9e–g illustrates the shape-based separation of *T. eccentrica* at the downstream outlets of the microchannel. Figure 9e shows the shape-based separation of *T. eccentrica* with ARs = 3 and 1.5, Fig. 9f shows the microalgae with ARs = 1.9 and 1.5, and Fig. 9g represents those with ARs = 2 and 1.35 using *T. eccentrica* of similar but different sizes. These experiments involved *T. eccentrica* with varying ARs, which deviated along distinct trajectories within the microchannel under the influence of TSAW, aligning with earlier observations for the polymer microparticles. Subsequently, the particles exited through different microchannel outlets, thereby demonstrating the feasibility of shape-based separation. These experimental results suggest the potential applicability of the proposed acoustofluidic shape-based separation method for biological samples in further studies.

## Conclusion

We proposed an acoustofluidic shape-based method to separate spherical and prolate micro-objects. The spherical PS microparticles were synthesized by the emulsifier-free emulsion polymerization method, and prolate ellipsoidal PS microparticles with varying aspect ratios were fabricated by uniaxial stretching using seed microspheres. The acoustofluidic device, composed of an SFIT deposited on a LiNbO_3_ substrate, was used to generate TSAWs with varying frequency. From the numerical simulation of wave scattering of spherical and prolate ellipsoids with varying aspect ratios, we discovered that asymmetric wave scattering induced counterrotating radiation torque on rotating prolate objects and aligned objects so that the major axis was parallel to the progressive wave propagation. These aligned prolate microparticles led to a reduced cross-sectional area perpendicular to the wave propagation, and the acoustic backscattering was reduced, resulting in a decreased magnitude of the radiation force. We achieved acoustofluidic separation of spherical and prolate PS microparticles, as well as spherical and peanut-shaped PS microparticles, of the same or similar volume. From the quantitative analysis of the separation efficiency, we confirmed the high purity and recovery rate of the separated spherical and peanut-shaped PS microparticles using the proposed acoustofluidic method. This pioneering paper on acoustofluidic shape-based separation focuses on prolate ellipsoids to thoroughly investigate the fundamental physics of prolate ellipsoids with respect to spheroids. We also validated the applicability of the proposed acoustofluidic method for bioparticle manipulation with nonspherical microalgae. We believe that the findings in the present study can provide valuable insights for future studies on the behaviors of irregularly shaped particles under the influence of an acoustic field.

### Supplementary information

Supplementary Material

Supplementary Movie S1
