# Supplementary material for: Acoustofluidic separation of prolate and spherical micro-objects
Source: Microsyst Nanoeng. 2024 Jan 11;10:6. doi: 10.1038/s41378-023-00636-7 (PMC10784511; doi:10.1038/s41378-023-00636-7)
Supplement: Supplementary file 1 — Supplementary Material [file 41378_2023_636_MOESM1_ESM.docx]

**Supplementary Material**

Acoustofluidic Separation of Prolate and Spherical Micro-Objects

Muhammad Soban Khan,^1^ Mushtaq Ali,^1^ Song Ha Lee,^1^ Keun Young Jang,^2^ Seong Jae Lee,^2^ and Jinsoo Park^1^

^1^Department of Mechanical Engineering, Chonnam National University, 77 Yongbong-ro, Buk-gu, Gwangju 61186, Republic of Korea

^2^Department of Polymer Engineering, The University of Suwon, 17 Wauan-gil, Bongdam-eup, Hwaseong, Gyeonggi 18323, Republic of Korea

Muhammad Soban Khan (sobankhan@jnu.ac.kr)

Mushtaq Ali (alimushtaq012@jnu.ac.kr)

Song Ha Lee (thdgk14@jnu.ac.kr)

Keun Young Jang (gywlslover@naver.com)

Seeing Jae Lee (sjlee@suwon.ac.kr)

*Correspondence: Jinsoo Park (jinsoopark@jnu.ac.kr, [T] +82-62-530-1672 [F] +82-62-530-1689)


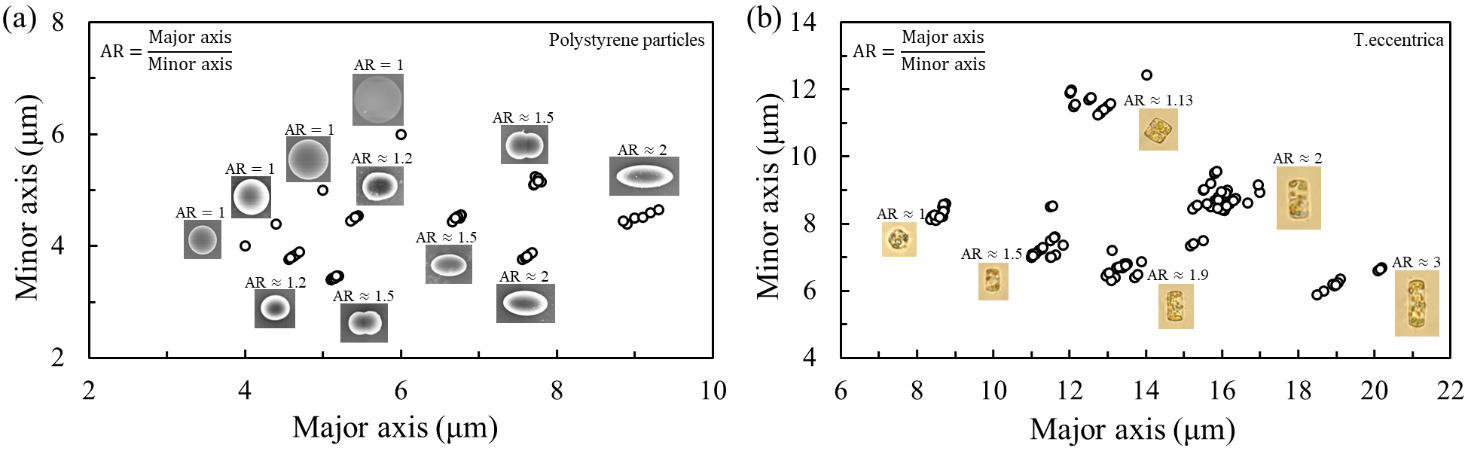


**Figure S1.** Statistics of the particle dimensions for each particle type used in the experiments (a) polystyrene particle and (b) T.eccentrica.

**Table S1.** Dimensions of all the particles used in the experiments


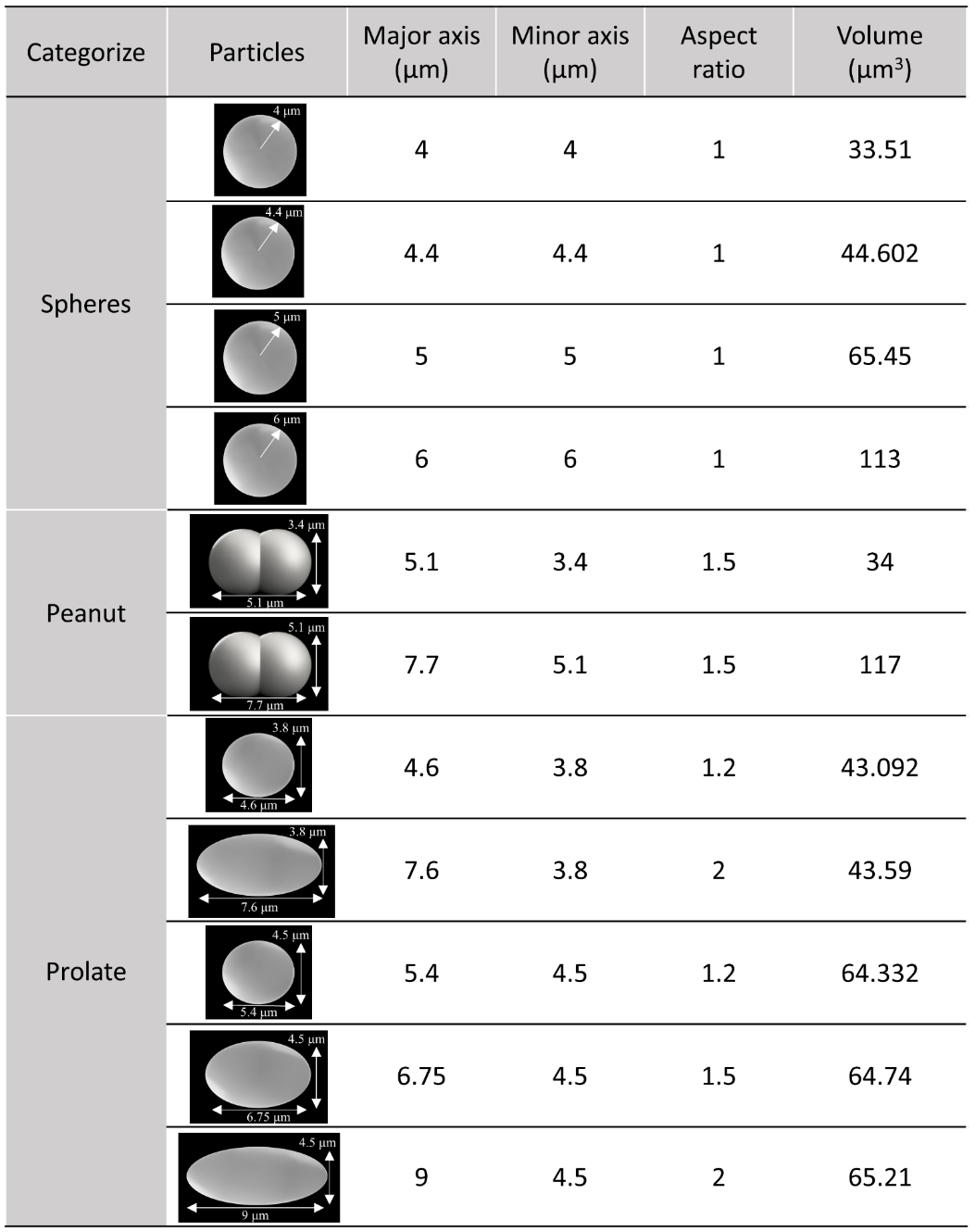


**Table S1.** Dimensions of all the particles used in the experiments.
